# Glicentin‐related pancreatic polypeptide inhibits glucose‐stimulated insulin secretion from the isolated pancreas of adult male rats

**DOI:** 10.14814/phy2.12638

**Published:** 2015-12-03

**Authors:** Lynda Whiting, Kevin W. Stewart, Deborah L. Hay, Paul W. Harris, Yee S. Choong, Anthony R. J. Phillips, Margaret A. Brimble, Garth J. S. Cooper

**Affiliations:** ^1^ School of Biological SciencesUniversity of AucklandAucklandNew Zealand; ^2^ The Maurice Wilkins Centre for Molecular BioDiscoveryNew Zealand; ^3^ Waikato Institute of TechnologyHamiltonNew Zealand; ^4^ School of Chemical SciencesUniversity of AucklandAucklandNew Zealand; ^5^ Department of SurgeryFaculty of Medical & Health SciencesUniversity of AucklandAucklandNew Zealand; ^6^ Centre for Advanced Discovery and Experimental TherapeuticsNIHR Manchester Biomedical Research CentreCentral Manchester University Hospitals NHS Foundation TrustManchesterUK; ^7^ The Institute of Human DevelopmentUniversity of ManchesterManchesterUK; ^8^ Department of PharmacologyMedical Sciences DivisionUniversity of OxfordOxfordUK

**Keywords:** GLP‐1, glucagon, GRPP, GSIS, proglucagon

## Abstract

Peptides derived from the glucagon gene *Gcg*, for example, glucagon and glucagon‐like peptide 1 (GLP‐1), act as physiological regulators of fuel metabolism and are thus of major interest in the pathogenesis of diseases, such as type‐2 diabetes and obesity, and their therapeutic management. Glicentin‐related pancreatic polypeptide (GRPP) is a further, 30 amino acid *Gcg‐*derived peptide identified in human, mouse, rat, and pig. However, the potential glucoregulatory function of this peptide is largely unknown. Here, we synthesized rat GRPP (rGRPP) and a closely related peptide, rat GRPP‐like peptide (rGRPP‐LP), and investigated their actions in the liver and pancreas of adult male rats by employing isolated‐perfused organ preparations. Rat GRPP and rGRPP‐LP did not affect glucose output from the liver, but both elicited potent inhibition of glucose‐stimulated insulin secretion (GSIS) from the rat pancreas. This action is unlikely to be mediated by glucagon or GLP‐1 receptors, as rGRPP and rGRPP‐LP did not stimulate cyclic adenosine monophosphate (cAMP) production from the glucagon or GLP‐1 receptors, nor did they antagonize glucagon‐ or GLP‐1‐stimulated cAMP‐production at either receptor. GRPP and GRPP‐LP may be novel regulators of insulin secretion, acting through an as‐yet undefined receptor.

## Introduction

**Figure 1:**
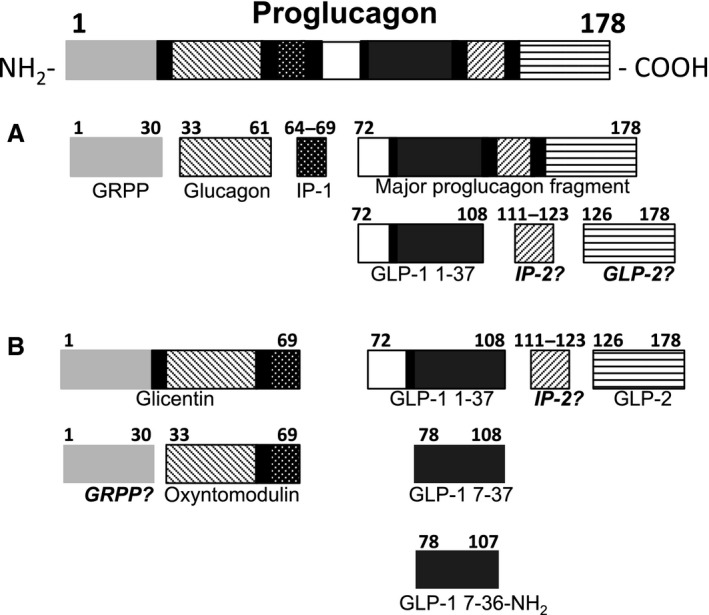
Theoretical processing of proglucagon in the pancreas and intestine. GRPP, glicentin‐related pancreatic polypeptide; IP‐1, intervening peptide; GLP‐1, glucagon‐like peptide 1; IP‐2, intervening peptide 2; GLP‐2, glucagon‐like peptide 2; modified from Holst (Holst 1997). Peptides labeled in italics have been presumed to be present but have not been identified in mammals.

Preproglucagon, derived from the *Gcg* gene, is the peptide precursor for several important endocrine hormones and shows a tissue‐specific processing pattern (Fig. 1). In intestinal L‐cells and neurons, proglucagon processing results in the liberation of glicentin, oxyntomodulin, GLP‐1, and glucagon‐like peptide‐2 (GLP‐2) (Holst 1997). By contrast, in the pancreatic *α*‐cells, proglucagon is processed to yield glucagon along with glicentin‐related peptide (GRPP) and an apparently nonbiologically active proglucagon fragment consisting of amino acids 72–158 (Patzelt and Schiltz 1984; Holst 1997). Glucagon, GLP‐1, GLP‐2, and oxyntomodulin are well studied and are known to have numerous important functions: either directly or indirectly, they each modulate glucose homeostasis (Myers et al. 1991; Magnusson et al. 1995; Stumpel et al. 1998; Jeppesen et al. 2001; Vahl et al. 2003). GLP‐1 is an incretin that has given rise to two new classes of antidiabetic agents: the GLP‐1 agonists and, indirectly, the dipeptidyl peptidase‐4 (DPP4) inhibitors. Glucagon is another potent *Gcg*‐derived glucoregulatory hormone whose main physiological role is to increase glucose output from the liver.

Considerably less is known of the actions of GRPP, which has been little studied to date. This 30‐amino acid peptide was originally purified from porcine pancreas (Thim and Moody 1982). It has also been identified in rat, mouse, and human islets along with insulin, glucagon, and amylin (Minerva et al. 2008; Stewart et al. 2011; Taylor et al. 2013). A further peptide, closely related to GRPP, GRPP‐like peptide (GRPP‐LP) was also identified in Wistar rat islets (Stewart et al. 2011). This molecule is similar to GRPP but differs in that GRPP‐LP has the carboxyterminal sequence—PNQINE (Table 1).

**Table 1:** Comparison of GRPP amino acid sequences derived from proglucagon

Gene ID	Species	Sequence	Sequence identity
gi¦204370	*Rattus norvegicus*	HAPQDTEENARSFPASQTEPLEDPDQINED	30/30 (100%)
rGRPP‐LP (Patzelt and Schiltz 1984)	*Rattus norvegicus*	HAPQDTEENARSFPASQTEPLEDP**N**QINE	28/30 (97%)
gi¦183270	*Homo sapiens*	**RSL**QDTEE**KS**RSF**S**ASQ**AD**PL**S**DPDQ**M**NED	20/30 (67%)

Comparison of GRPP and GRPP‐LP from rat preproglucagon and GRPP from human preproglucagon. Residues differing from the GRPP‐like peptide are bold and underlined.

The identification of both GRPP and GRPP‐LP as potential products from the proglucagon precursor suggests that both peptides could be secreted from the *α*‐cell along with glucagon. As yet, there are currently no immunological tools or antagonists for GRPP or GRPP‐LP, meaning that understanding of the expression and function of this peptide is in its infancy. Glucagon fulfills a major physiological role in maintaining glucose homeostasis through its effects in the liver. The effects of GRPP and GRPP‐LP on liver have yet to be investigated.

Both GLP‐1 and glucagon are reported to act on the pancreatic *β*‐cell to enhance glucose‐stimulated insulin secretion (GSIS) (Metz et al. 1983; Zielmann et al. 1985; Holst et al. 1987; Shima et al. 1988; Goke et al. 1989; Ishizuka et al. 1991; Jia et al. 1995; Vahl et al. 2003). However, we know of only one study reporting the effects of GRPP on GSIS. In that study, which employed a local circulation preparation of the canine pancreas, the administration of human GRPP and human glicentin 1–16 reportedly caused slight increases in plasma insulin and decreases in plasma glucagon (Ohneda and Ohneda 1988). This tantalizing finding suggests that GRPP might also be an unrecognized regulator of GSIS. We, therefore, questioned whether rGRPP and rGRPP‐LP can act like GLP‐1 to modulate GSIS in the rat pancreas and/or, like glucagon, to modulate glucose production in the liver.

## Materials and Methods

### Synthesis of rGRPP and rGRPP‐LP

Peptides were synthesized by solid‐phase peptide synthesis, using the 9‐fluorenylmethoxycarbonyl/tertiary‐butyl (Fmoc/^t^Bu) method on a 0.1‐mmol scale. Briefly, aminomethyl resin was prepared as described (Harris et al. 2011), functionalized with the appropriate linker, and the peptide elongated using a microwave peptide synthesizer (CEM, NC) as previously described (Harris et al. 2008). The peptides were cleaved from the resin with concomitant removal of side‐chain protecting groups with 94% trifluoroacetic acid, 1% triisopropylsilane, 2.5% water, and 2.5% ethane dithiol (v/v/v/v) for 2–3 h, precipitated with cold diethyl ether, isolated by centrifugation, dissolved in 50% aqueous acetonitrile containing 0.1% trifluoroacetic acid, and lyophilized. Crude peptides were purified by semipreparative reversed phase HPLC. Fractions containing the pure peptide were identified by electrospray mass spectrometry and/or HPLC, pooled, and lyophilized. All peptides were >95% purity as judged by integration of the HPLC chromatogram at 210 nm, and peptide masses were confirmed by electrospray mass spectrometry.

### Animals

All studies were performed under approval of the University of Auckland Animal Ethics Committee and were consistent with the ARRIVE guidelines (Kilkenny et al. 2012). Male Sprague‐Dawley rats (220–300 g) were obtained from a breeding colony in the Integrated Physiology Unit, School of Biological Sciences at the University of Auckland. Animals were housed in standard environmental conditions under a 12‐hour light/dark cycle and a temperature of 20 ± 1°C. They were maintained on Teklad TB 2018 (Harlan, UK) rat chow and tap water ad libitum.

### Isolated liver perfusion

Under general anesthesia (isoflurane 2–5%; 2 L min^−1^ O_2_ via nasal cone), nonfasted rats underwent laparotomy between 8:00 and 9:00 am on the experimental day. The liver was removed and quickly connected to a custom‐made, temperature‐controlled, organ perfusion system, whereby it was perfused (in nonrecirculating mode) in a manner similar to that reported previously (Englisch et al. 2000; Whiting et al. 2013). Livers were perfused at a rate of 2 mL g^−1^ min^−1^ with perfusion media containing final concentrations (in mmol L^−1^): NaCl, 128; NaHCO_3_, 23.9; KCl, 6.0; CaCl_2_, 2.4; MgSO_4_, 1.18; D‐glucose, 5; 3‐(‐*N*‐morpholino)propane sulfonate, 1.29; with fatty‐acid‐free BSA, 0.5% (w/v) at pH 7.4 gassed with O_2_:CO_2_ 95:5% (v/v) at 37°C. Each liver was stabilized for 40 min: thereafter, hormone‐containing solutions or vehicle (buffer only) was delivered via a side‐arm infusion. Hormones were also investigated with regard to their ability to attenuate epinephrine‐stimulated hepatic glucose output (Epinephrine, Sigma‐Aldrich, St. Louis, MO). Epinephrine was added 30 min after the addition of hormones was commenced. All livers were viable over the period of the perfusion as indicated by their prompt responsiveness to glucagon (Glucagen; Novodisk, Novo Allé, Denmark), which was added alone to the buffer at 60 min.

### Isolated pancreas perfusion

General aspects of the preparation for the isolated pancreas perfusions were the same as those for the liver perfusion studies (above). Particular aspects of the preparation for the pancreas perfusion studies were as follows. After laparotomy, the pancreas and duodenum were removed en bloc and perfused (in nonrecirculating mode) consistent with our previous report (Buchanan et al. 2001). Each pancreatic preparation was quickly connected to the custom‐made, temperature‐controlled, organ perfusion system and perfused at 3 mL min^−1^ with oxygenated perfusion buffer (containing at final concentrations in mmol L^−1^): NaCl, 112; NaHCO_3_, 29.3; KCl, 4.4; CaCl_2_, 2.4; MgSO_4_, 1.2; D‐glucose, 5.0; with BSA, 0.5% (w/v), Dextran 4% (w/v) at pH 7.4 gassed with O_2_:CO_2_ 95:5% (v/v) at 37°C. Peristalsis of the duodenum was visible throughout the entire perfusion period as an indicator of the preparation's viability. Each pancreas was stabilized for 30 min before hormones or vehicle (buffer only) was delivered via a side‐arm infusion. The human GLP‐1 7–36 was obtained from Abcam (Cambridge, UK).

### Measurement of organ perfusion parameters

Insulin was measured in perfusate from the pancreas preparation using an ELISA (Merck Millipore, Darmstadt, Germany) and glucagon using an RIA (Merck Millipore, Darmstadt, Germany): however, concentrations of the latter were uniformly below the minimum detectable concentration (data not shown). Glucose and lactate concentrations were measured in both organ preparations using a YSI2300 Stat glucose and lactate analyzer (Yellow Springs, Ohio). Portal pressure was measured in isolated‐perfused liver preparations in mmH_2_O via a T‐tube located 10 cm upstream from the preparation.

### Transfection of Cos 7 cells

Cos 7 cells were maintained and subcultured as previously described (Bailey and Hay 2006) and plated at a density of 15,000 cells per well into 96‐well plates 1 day prior to transfection. Expression clones for the human GLP‐1 receptor (hGLP‐1R) and human glucagon receptor (hGL‐R) were kindly provided by Professor Patrick Sexton (Monash University, Melbourne, Australia) and were here transfected using polyethylenimine as previously described with 0.25 *μ*g of plasmid DNA per well (Bailey and Hay 2006). Cyclic AMP assays were performed 48 h after transfection (Bailey and Hay 2006). The human GRPP was obtained from Bachem (Bubendorf, Switzerland).

### Cyclic AMP stimulation of transfected Cos 7 cells

Transfected cells were serum‐deprived in DMEM containing 1 mmol L^−1^ IBMX and 0.1% (w/v) bovine serum albumin (BSA) for 30 min. GRPP, GRPP‐LP, glucagon, or GLP‐1 7–36 at different concentrations were added to wells, along with forskolin (50 μmol L^−1^, Tocris Bioscience, Wiltshire, UK) which was included as a positive control on each plate. Plates were incubated at 37°C for 10 min. For antagonist experiments, GRPP was preincubated with the cells for 5 min before glucagon or hGLP‐1 7–36 were added at indicated concentrations. Media were then aspirated from the wells and reactions terminated by addition of ice‐cold absolute ethanol. Ethanol was then evaporated to dryness and cell extracts re‐suspended in 45 *μ*L cAMP assay buffer: cAMP concentrations were determined using ALPHA‐screen assay kits (Perkin Elmer, Massachusetts) as previously described (Gingell et al. 2010).

### Glucose stimulation of isolated rat islets

Rat islets were isolated by the collagenase digestion method. For each experiment, 2–4 rats were used. Rats were killed under anesthetic (isoflurane 2–5%; 2 L min^−1^ O_2_ via nasal cone) and collagenase solution (collagenase type 4, 350 *μ* mg^−1^; Bioconcept, Switzerland) at 0.05 mg mL^−1^ infused into the common bile duct after occlusion of the distal end just proximal to the duodenum. Pancreata were then excised, and incubated in a waterbath at 37°C for 30 min. At the end of digestion, the tissue was shaken for 1 min and washed with HBSS. Tube contents were filtered through a 500 *μ*m plastic mesh to discard any undigested tissue and washed again in HBSS. The resulting pellet was filtered onto a 70‐μmol L^−1^ strainer, and islets were then transferred into RPMI 1640 medium containing 10% BSA and 1% of penicillin/streptomycin and handpicked. Islets were plated at 10 islets/well and cultured in RPMI media for 48 h. Insulin secretion was measured by static incubation using KRB (in mmol L^−1^: NaCl, 129; NaHCO_3_, 5; KCl, 4.8; KH2PO4, 1.2; MgSO_4_, 1.2; HEPES, 10; CaCl_2_, 2.5; 0.1% (w/v) BSA. The incubation experiments were started with a 30 min preincubation of the islets in KRB with 2.8 mmol L^−1^ glucose. Islets were then incubated for 1 h each in KRB supplemented with glucose (2.8 and 16.7 mmol L^−1^) and different concentrations of hGRPP (1–100 nmol L^−1^) or 20 nmol L^−1^ GLP‐1 7–36. The medium was collected for each incubation period and insulin content of islets were extracted using 0.18 mol L^−1^ HCl‐70% ethanol. Insulin concentration was measured by ELISA technique (Mercodia, Sweden). Experiments were repeated three times with three incubations per condition in each experiment.

### Data analysis and statistics

Maximum pancreatic first‐phase insulin secretion in the presence of hormone infusion was contrasted with corresponding control values by unpaired Welch's *t*‐tests for preplanned comparisons, to allow for unequal variance between groups. Linear mixed‐effects models were fitted by restricted maximum likelihood (LME‐REML) to data obtained from each indicated phase of isolated perfused–organs studies, using S‐PLUS version 8.2 (TIBCO, Spotfire). Calculated *P*‐values <0.05 are presented in the article and reflect the likelihood ratio between two models, the effect in question (treatment, time, or treatment:time) against the model without the effect in question. Insulin secreted from isolated islets was expressed as a percentage of insulin content and compared using one‐way ANOVA (GraphPad Prism version 6.0: GraphPad Software Inc., San Diego, CA). Cyclic AMP data have been normalized to the response obtained to forskolin and fitted to obtain concentration–response curves using a three‐parameter logistic equation (GraphPad Prism version 6.0: GraphPad Software Inc., San Diego, CA): pEC_50_ values were obtained from the concentration–response curves and compared using one‐way ANOVA. All data are presented as mean ± SEM of three to four independent experiments.

## Results

### Rat GRPP and rGRPP‐LP do not modify glucose production in the perfused rat liver

**Figure 2:**
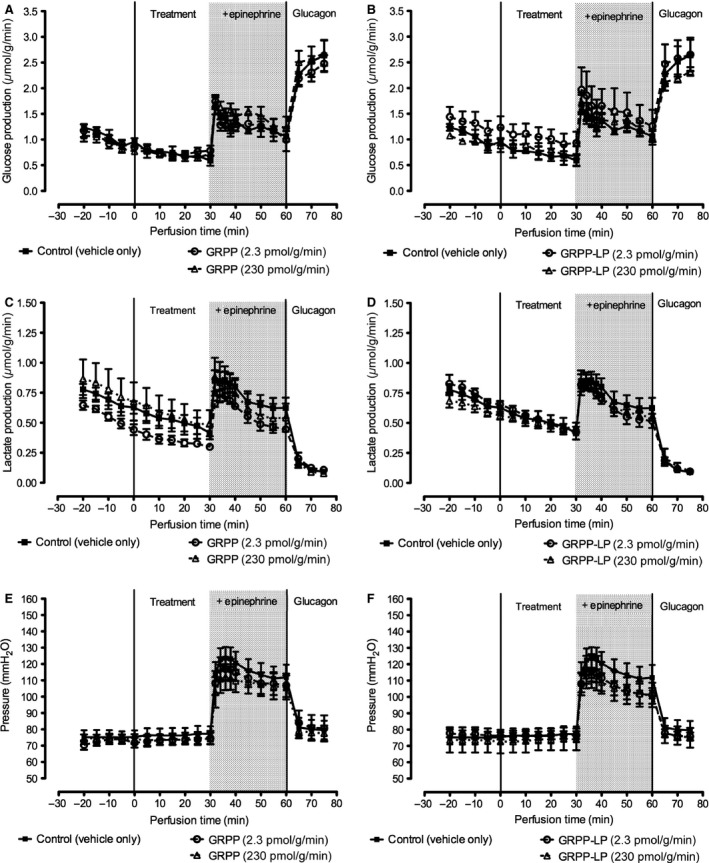
Effect of rGRPP and rGRPP‐LP on glucose output, lactate output, and portal pressure in the isolated perfused rat liver. Livers from Sprague‐Dawley rats were isolated and perfused with rGRPP (left panels: 2.3 or 230 pmol g^−1^ min^−1^, *n* = 5 and *n* = 3, respectively), rGRPP‐LP (right panels: 2.3 or 230 pmol g^−1^ min^−1^, *n* = 5 and *n* = 3), or vehicle (all panels: control, *n* = 5). Livers were perfused with hormones or vehicle from 0 to 60 min with 0–30 min as the basal period. At 30 min, the addition of epinephrine (100 pmol g^−1^ min^−1^, 30–60 min) increased glucose production (A–B), lactate production (C–D), and portal pressure (E–F). Neither dose of rGRPP nor rGRPP‐LP modified these parameters compared with control values. Glucagon (2.3 pmol g^−1^ min^−1^) alone was administered from 60 to 75 min to verify organ viability.

The effects of hormones on glucose production, lactate production, and portal pressure in perfused livers are presented in Figure 2. Glucose output decreased slightly in all 6 groups over the 30‐min basal period, but there were no significant differences between any of the 6 groups in this phase. At *t *=* *30 min, the addition of 100 pmol g^−1^ min^−1^ epinephrine evoked an increase in glucose output in all groups that was not modified by rGRPP or GRPP‐LP. At *t *=* *60 min, infusion of vehicle, rGRPP, rGRPP‐LP, and epinephrine were terminated, and an infusion of 2.3 pmol g^−1^ min^−1^ glucagon commenced to check viability and the potential for glucagon receptor‐responsiveness. Glucagon increased glucose output by at least 2.5‐fold. There were no significant differences in the glucose responses of livers to glucagon between those perfused with any treatment compared with the control group. During the basal period, lactate output and portal pressure were not different between all groups verifying viability of the preparations. Epinephrine induced the expected elevation of lactate release and portal pressure in all groups without differences between the groups. During glucagon infusion, lactate output decreased to below basal levels and portal pressure returned to basal levels, consistent with the stimulation of gluconeogenesis—further indications of viability (Exton and Park 1968). There were no significant differences in lactate and portal pressure between livers perfused with any treatment compared with the control group.

### Rat GRPP and rGRPP‐LP inhibit GSIS in the isolated perfused rat pancreas

**Figure 3:**
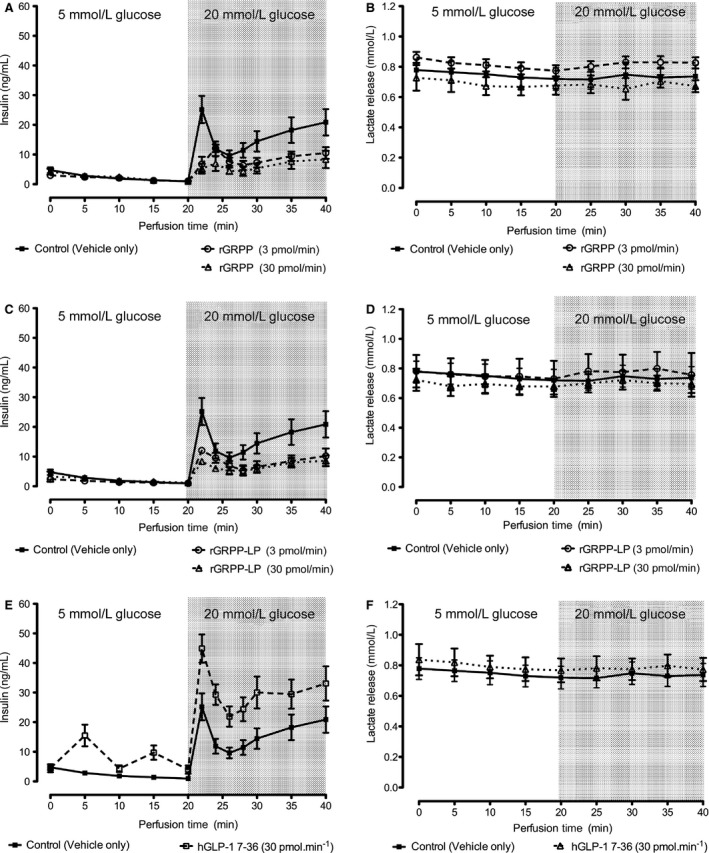
Effects of proglucagon products on insulin secretion in the isolated‐perfused rat pancreas. Pancreases isolated from Sprague‐Dawley rats were perfused with 5‐mmol L^−1^ glucose and either 30 pmol min^−1^ hGLP‐1 7–36 (A, B), 3 or 30 pmol min^−1^ rGRPP (C,D), 3 or 30 pmol min^−1^ rGRPP‐LP (E, F), or vehicle (A–F, solid lines). Glucose was increased to 20 mmol L^−1^ at 20 min (shaded area): *n* = 5/group. hGLP‐1 7–36 increased insulin secretion at 5‐mmol L^−1^ glucose and at 20‐mmol L^−1^ glucose compared with the control (A). By contrast, rGRPP and rGRPP‐LP all inhibited GSIS (C,E). Lactate output (B, D, F) remained at a constant level throughout the perfusion in all groups, and there were no significant differences between the groups.

No significant difference in insulin secretion was observed during the perfusion of 5‐mmol L^−1^ glucose between pancreases perfused with rGRPP, rGRPP‐LP, and glucagon compared with the control group (Fig. 3). When hGLP‐1 7–36 was perfused, there was a pulsatile change in insulin secretion over the 20 min during 5‐mmol L^−1^ glucose perfusion. At 20 min, glucose was increased to 20 mmol L^−1^ in all groups. The control (vehicle) group demonstrated the characteristic biphasic increase in insulin secretion, and this response was substantially elevated by infusion of the incretin hGLP‐1 7–36. The main effect of GLP‐1 7–36 occurs during the first phase, whereas during the second phase of GSIS, the curves for this hormone and for the control infusion are essentially parallel and this is reflected in the nonsignificant *P* value for the time:treatment (Table 2). In contrast, rGRPP, rGRPP‐LP, and glucagon significantly suppressed GSIS during the 20‐min perfusion with 20‐mmol L^−1^ glucose (Fig. 3). Whereas the second phase progressively increased in the control infusion, these peptides exert an ongoing suppression of insulin secretion throughout the second phase, and these effects are reflected in the strongly significant time‐dependent *P*‐values for each of these infusions (Table 2). Lactate was also measured in the perfusate at 5‐min intervals, and no significant differences were found between any of the groups.

**Table 2:** Effects of infusion of *Gcg*‐derived peptide hormones on the first and second phases of insulin secretion from isolated perfused rat pancreas preparations

Peptide infusion	First phase	Second phase		
		Time	Treatment	Time:Treatment
GLP‐1 7‐36, 30 pmol^−1^ min^−1^	0.015	0.0005	NS	NS
rGRPP, 30 pmol^−1^ min^−1^	0.009	<0.0001	NS	0.0039
rGRPP, 3 pmol^−1^ min^−1^	0.011	<0.0001	NS	0.0063
rGRPP‐LP, 30 pmol^−1^ min^−1^	0.019	<0.0001	0.047	0.0006
rGRPP‐LP, 3 pmol^−1^ min^−1^	0.044	<0.0001	NS	0.018

Calculated *P*‐values derived from Welch's *t*‐tests for first‐phase insulin secretion data, and linear mixed‐effects models (LMEM) of the form (Insulin Secretion ~ [Time + Treatment + Time:Treatment]) fitted by restricted maximum likelihood to data describing the second phase of insulin secretion following infusion of peptide solutions. Time values used for these calculations were at *t *=* *22 min for the first phase and from *t *=* *26 min until the end of the infusion at *t *=* *40 for the second phase. NS, not significant (i.e., *P *>* *0.05) comparing insulin secretion in control pancreases with that in pancreases perfused with hGLP‐1 7–36 (30 pmol min^−1^), rGRPP (3 or 30 pmol min^−1^), or rGRPP‐LP (3 or 30 pmol min^−1^).

### Human GRPP does not affect insulin secretion in rat islets

**Figure 4:**
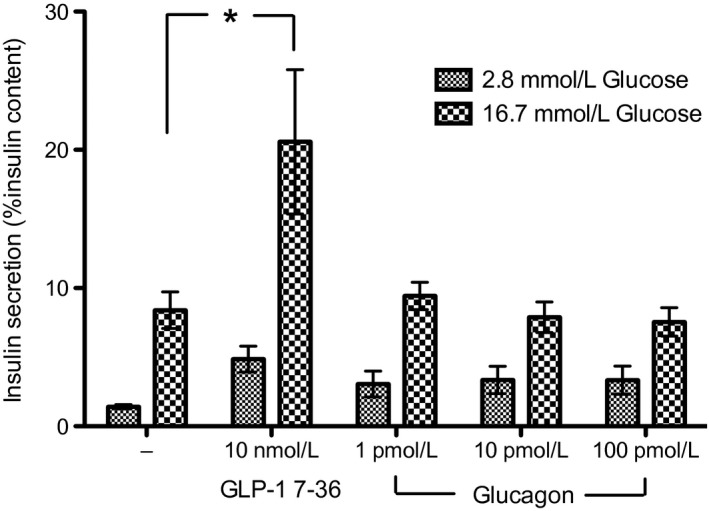
Human GRPP and glucagon do not affect GSIS in isolated rat islets. Effects of hGRPP on basal (2.8 mmol L^−1^ glucose) and stimulated (16.7 mmol L^−1^ glucose) insulin secretion. Each bar represents mean ± SEM from 8 to 9 incubations of 10 islets/well performed in three separate experiments. GLP‐1 7–36 increases GSIS compared with untreated islets (*P < 0.05 compared with control). In contrast, hGRPP at all concentrations did not affect basal or stimulated insulin compared with untreated islets.

Incubation of islets in 16.7‐mmol L^−1^ glucose alone increased insulin secretion compared with insulin secreted when islets were incubated in 2.8‐mmol L^−1^ glucose (Fig. 4). As expected, 20‐nmol L^−1^ hGLP‐1 increased insulin secretion in rat islets at both 2.8‐mmol L^−1^ and 16.7‐mmol L^−1^ glucose compared with the corresponding controls (Fig. 4). The addition of hGRPP at concentrations of 1, 10, and 100 nmol L^−1^ did not affect insulin secretion at 2.8‐mmol L^−1^ glucose and at 16.7‐mmol L^−1^ glucose compared with the corresponding controls (Fig. 4).

### Rat GRPP and rGRPP‐LP do not activate or antagonize cAMP production via GL‐R or GLP‐1R in transfected Cos 7 cells

**Figure 5:**
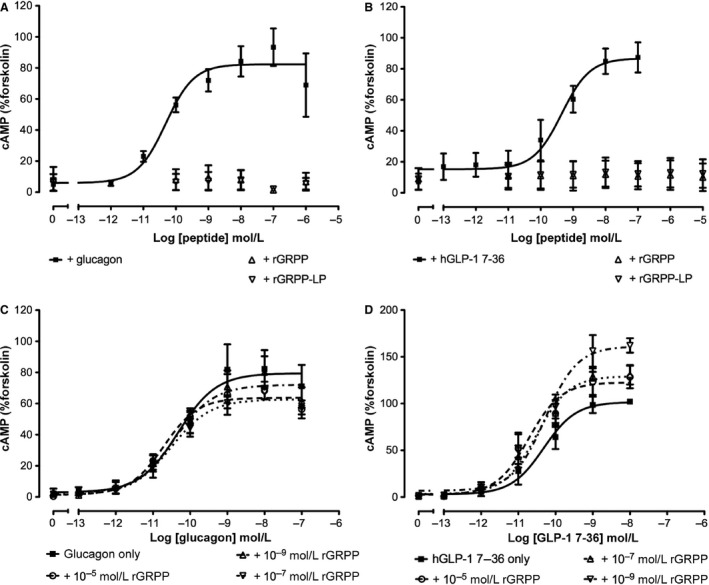
GRPP or GRPP‐LP does not stimulate or inhibit cAMP production through GL‐R and GLP‐1R. Glucagon stimulated cAMP production (A) and hGLP‐1 7–36 stimulated cAMP production (B) in Cos 7 cells transfected with their respective receptors, hGL‐R and hGLP‐1R. In contrast, rGRPP and rGRPP‐LP did not stimulate cAMP production at either receptor (A, B). Rat GRPP did not modify glucagon‐stimulated (C) or GLP‐1 7–36‐stimulated (D) cAMP production in Cos 7 cells transfected with hGL‐R or hGLP‐1R, respectively.

The receptor for GRPP is currently unknown. We investigated the possibility that GRPP or GRPP‐LP might act via either the GLP‐1R or GL‐R in transfected Cos 7 cells. Glucagon at the hGL‐R and hGLP‐1 7–36 at the hGLP‐1R produced concentration‐dependent increases in cAMP (Fig. 5). The mean pEC_50_ (±SEM) for glucagon was 10.30 (0.14, *n* = 4) and for hGLP‐1 7–36 it was 9.59 (0.43, *n* = 3): by contrast, neither rGRPP nor rGRPP‐LP stimulated cAMP production at either receptor (Fig. 5). Furthermore, there were no shifts in the GLP‐1 7–36 or glucagon concentration–response curves in the presence of rGRPP (*P *>* *0.05, Fig. 5). The hGL‐R exhibits 82% identity to the rat receptor (Lok et al. 1994; MacNeil et al. 1994). Furthermore, RAMP2 has an association with the GL‐R receptor (Christopoulos et al. 2003; Weston et al. 2015). In an effort to determine whether RAMP2 is required for hGL‐R activation with hGRPP, Cos 7 cells were transfected with hGL‐R with the addition of a vector or RAMP2. However, in this preliminary experiment, hGRPP did not stimulate cAMP production at hGL‐R alone or in the presence of RAMP2 (data not shown).

## Discussion

GRPP and GRPP‐LP inhibited the initial first phase of GSIS in the isolated rat pancreas, and this inhibition was additionally sustained in the second phase. All pancreata were viable over the period of the perfusion as indicated by a lack of any statistically significant change in lactate output between the groups. This finding is noteworthy as it offers the possibility that GRPP and GRPP‐LP could play physiological roles in the regulation of GSIS, along with the other known proglucagon‐derived products, glucagon, and GLP‐1.

GLP‐1 7–36 evoked a significant increase in GSIS in both the rat pancreas and isolated islets. Although it is recognized that GLP‐1 7–36 amide and GLP‐1 7–37 are the more likely forms produced, it has been reported that the carboxyterminal of GLP‐1 does not have a significant effect on insulin secretion in the perfused pancreas (Suzuki et al. 1989).

Although GRPP and rat GRPP‐LP have been identified in islets, it has yet to be established whether GRPP and GRPP‐LP are secreted from *α*‐cells in response to physiological stimuli, such as glucose or arginine. Glucagon is secreted from the *α*‐cells of the pancreas in response to a decrease in blood glucose and is also stimulated by arginine (Weir et al. 1976; Dunbar and Brown 1984; Myers et al. 1991). Given that GRPP and GRPP‐LP are also processing products from the same precursor, it could be proposed that these peptides are also secreted in response to low glucose. However, this proposal does not easily fit with the present findings that the addition of GRPP and GRPP‐LP inhibits GSIS. Furthermore, neither peptide inhibited insulin secretion against a low glucose background. According to the theoretical model of proglucagon, processing for each tissue type is as put forward by Holst (Fig. 1). GRPP could also be present in the intestinal L‐cells along with oxyntomodulin; however, this has yet to be explored.

Previously, porcine GRPP was found to have a small effect on glucagon and insulin secretion in the canine pancreas, whereby insulin was possibly increased and glucagon decreased (Ohneda and Ohneda 1988). Furthermore, human glicentin 12–69 (which encompasses both GRPP and glucagon sequences) enhanced GSIS and inhibited glucagon secretion in the perfused canine pancreas (Yanaihara et al. 1985). The precise experimental conditions such as amount of glucose and experimental model used may have influences on the different outcomes; however, the reason for the opposing actions of GRPP between these studies could be due to species differences. Interestingly, in our study, GRPP did not affect GSIS in isolated rat islets. GRPP is not fully conserved between species; hGRPP has 67% of its amino acids conserved with rat (Table 1). The lack of effect of hGRPP in rat islets could relate to this. Nevertheless, ours is the first matched‐species study in the whole pancreas and shows that rGRPP has an effect on insulin secretion: this finding warrants further investigation.

GRPP and GRPP‐LP may inhibit insulin secretion indirectly, perhaps by influencing other islet‐derived hormones known to modulate hormone secretion from the *β*‐cell. For example, somatostatin (SST) is an influential peptide produced in pancreatic‐islet *δ*‐cells that inhibits both insulin and glucagon secretion. In humans, infusion of SST decreased arginine‐stimulated plasma insulin and glucagon levels in a dose‐dependent manner (Skamene and Patel 1984). In the isolated‐perfused rat pancreas, perifused rat islets, and cultured islets, SST inhibits GSIS as well as arginine‐stimulated glucagon secretion (Norfleet et al. 1975; Fujimoto 1981; Bolaffi et al. 1990). Recently, an additional peptide of the SST preprohormone, neuronostatin, has been identified and also found to inhibit GSIS in rat islets (Salvatori et al. 2014). Furthermore, the authors found that this effect was more likely a result of neuronostatin directly acting on the *α*‐cell as administration of neuronostatin in both islets, and the *α*‐cell line *α*TC1‐9 increases glucagon secretion, but failed to affect insulin secretion in the *β*‐cell line INS 832/13 where *α*‐cells are not present (Salvatori et al. 2014). Other islet hormones influence the secretion of SST. For example, exogenous glucagon at supraphysiological levels in the presence of glucose increases SST in isolated rat islets (Patel et al. 1979; Dolais‐Kitabgi et al. 1981). Therefore, it is possible that the administration of rGRPP or rGRPP‐LP in the perfused rat pancreas could increase SST or neuronostatin secretion, inhibiting GSIS. However, there is as yet no direct evidence for this putative mechanism.

These current findings raise the possibility that there is a more complex feedback system present in islets, whereby islet‐derived peptide hormones could influence each other's production and effects. It is important to acknowledge that although physiological events such as GSIS are similar between rodent and human, the functional organization within the islet to achieve this broad similarity is different between rodent and man (Barker et al. 2013; Caicedo 2013). Human islets contain more *α*‐cells than mouse islets and, unlike rodents, these cells are aligned along blood vessels with no particular order or arrangement within the islet (Brissova et al. 2005, 2015; Cabrera et al. 2006). As a result, the human islet contains a heterogeneous cell population through which blood flows. It is, therefore, unlikely that there is a hierarchy in the sequence in which the different endocrine cells are perfused in the human islet. This is thought to predispose human islets to strong paracrine interactions (Barker et al. 2013). These anatomical arrangements are likely to have consequences for the effects of GRPP in human islets. Therefore, it is also of interest to investigate the role human GRPP plays in the human pancreas. Further investigations are needed to more fully comprehend how islet hormones influence each other with the ultimate goal of understanding how these various influences might sum to modulate GSIS in health and disease.

Given that we found an inhibitory effect of rGRPP and rGRPP‐LP on GSIS in the isolated perfused rat pancreas, and glucagon and GLP‐1 increase cAMP production through binding to and activating signaling pathways via GLP‐1R and GL‐R, respectively, we investigated the activity of rGRPP and rGRPP‐LP at these receptors to search for a possible mechanism for their action. However, neither rGRPP nor rGRPP‐LP activated these receptors when added alone: nor did rGRPP block cAMP production evoked by glucagon or GLP‐1 7–36. It is possible that GRPP and GRPP‐LP could activate another pathway via these receptors. Further studies are needed with rat receptors and radioligand binding assays to confirm the lack of interaction at the GL‐R and GLP‐1R. However, the lack of effect of GRPP and GRPP‐LP in the liver supports a lack of activation of the glucagon receptor.

There is also the possibility that GRPP and GRPP‐LP may require a receptor activity‐modifying protein (RAMP). In transfected HEK‐293 cells, GL‐R was found to interact specifically with RAMP‐2, and not other RAMPs, leading to translocation of RAMP‐2 to the cell surface as well as an increase in total RAMP‐2 levels (Christopoulos et al. 2003; Weston et al. 2015). The specific pairing of GL‐R with only one of the RAMPs and the known impact for their diversity on function, particularly on the calcitonin family of receptors, suggests that this may be biologically significant (Christopoulos et al. 2003). However, a preliminary experiment where hGRPP was tested in Cos 7 cells transfected with hGL‐R and with the addition of a vector or RAMP2 indicates that the modification of this receptor by RAMP2 does not allow activation by GRPP. Further, it suggests that the lack of effect of rGRPP on cAMP production at the human receptors is not due to species differences.

Rat GRPP and rGRPP‐LP may exert their effects through an as‐yet unknown receptor such as one of the many orphan G‐protein‐coupled receptors expressed in islets but whose effects on islet hormone secretion are largely unknown due to a lack of currently available pharmacological tools (Nambi and Aiyar 2003; Amisten et al. 2013). Two possible mechanisms that have been found to enhance GSIS and both involve an increase in intracellular Ca^2+^ (Ahren 2009). The activation of the receptor‐coupled enzyme phospholipase C (PLC) leads to the production of diacylglycerol (DAG), which liberates Ca^2+^ from intracellular storage sites, thereby increasing exocytosis of insulin. Second, increased intracellular levels of cAMP activate protein kinase A (PKA), which activates kinases that are important in exocytosis (Ahren 2009). It is possible that GRPP and GRPP‐LP are capable of inhibiting either of these pathways through any of these orphan G‐protein‐coupled receptors.

GRPP and GRPP‐LP inhibited GSIS in the rat pancreas. This finding is significant and leads to the question as to whether GRPP and/or GRPP‐LP are physiological regulators of glucose homeostasis. This requires further exploration. The importance of controlling GSIS in diabetes together with the potential of rGRPP and rGRPP‐LP to regulate GSIS warrants further investigation into the putative effects and mechanisms of GRPP.

## Conflict of Interest

None declared.
